# Vascular endothelial growth factor expression and pathological changes in the local tissue of facial hemangiomas following injections with pure alcohol

**DOI:** 10.3892/ol.2014.2802

**Published:** 2014-12-17

**Authors:** ZHAO-JUN FU, CHUN-MING LI, TAI-HE WANG, ZHU-LING JIANG, ZHAO-CHEN FU

**Affiliations:** 1 Department of Aviation Diseases, General Hospital of Air Force, Beijing 100142, P.R. China; 2 Department of Oral and Maxillofacial Surgery, The Second Affiliated Hospital of Harbin Medical University, Harbin, Heilongjiang 150001, P.R. China

**Keywords:** hemangioma, vascular endothelial growth factor, pure alcohol, enzyme-linked immunosorbent assay

## Abstract

The aim of the present study was to investigate the association between the formation of hemangioma and the expression of vascular endothelial growth factor (VEGF) following local injections of pure alcohol in patients exhibiting hemangioma. Ten healthy subjects (control group) and 10 hemangioma patients (treatment group) were included in the study population, with the hemangioma patients receiving one injection of pure alcohol. The VEGF levels were evaluated in the treatment and control group subjects prior to and following the injection using enzyme-linked immunosorbent assay; furthermore, local tissue was excised to perform pathological analysis one week after the injections. The VEGF levels of the healthy group were identified to be significantly lower when compared with those of the treatment group prior to the injections (P<0.01) and one week after the injections (P<0.01), however, were not significantly different when compared with the treatment group one month after the injections (P>0.01). Therefore, serum VEGF concentrations in the peripheral blood may be a clinical indicator of the efficacy of clinical treatment and aid with determination of the prognosis.

## Introduction

The causal factors for hemangiomas have remained unclear until recently. However, it is widely hypothesized that vascular endothelial growth factor (VEGF) is a major factor in the formation of hemangiomas. Shima *et al* (1) proposed that a shortage of oxygen initially prompts an organism to produce VEGF (1), and VEGF is a type of vessel growth factor, which stimulates the formation of hemangiomas and has previously been investigated in detail (2). Its primary biological functions are as follows: i) Selectively enhancing vascular endothelial cell proliferation and promoting the formation of blood vessels; and ii) participating in the mechanism that increases the permeability of blood vessels, particularly microvessels (3,4). By facilitating the ability to exude large molecules of plasma from a vessel into the surrounding tissue, VEGF promotes the growth of tumor cells, accelerates the formation of novel blood vessels and provides nutrition for these novel vessels (5).

There are various treatment methods for hemangiomas, including surgery, laser treatment, cryotherapy, partial radiation therapy and local injections. Local injections of therapeutic agents are administered directly into the blood vessels within the hemangioma. The agents commonly administered in clinical practice include the following: Corticosteroids, 5% morrhuate sodium, pingyangmycin and pure alcohol, amongst others. Pure alcohol is a traditional Chinese medicinal compound and its primary ingredients are gal, alum, glycerin, chlorobutanol and stabilizer compounds. Pure alcohol damages the cells of the endothelium of a hemangioma, resulting in clumping of the red blood cells, protein coagulation and thrombosis (6). These effects result in thrombus formation in the lumina of hemangiomas. Therefore, the method of injecting pure alcohol is simple, there is less damage to the patient and the range of applications is wide.

Enzyme-linked immunosorbent assay (ELISA) was used to determine the concentrations of serum VEGF in 10 healthy subjects and 10 hemangioma patients. The 10 hemangioma patients were injected with pure alcohol one week prior to the collection of blood samples, and eight of the hemangioma patients were subsequently injected with pure alcohol one month later. The association between VEGF and the morbidity resulting from hemangiomas was examined. Furthermore, the clinical efficacy of the method of injecting pure alcohol into hemangiomas was observed. In addition, the clinical efficacy of all of the treatment methods for hemangiomas that are adopted at the Department of Oral and Maxillofacial Surgery, the Second Affiliated Hospital of Harbin Medican University (Harbin, China), including laser therapy and surgical removal, are provided based on the experiments that are presented in the current study.

## Materials and methods

### Experimental principles

The present study employed the double antibody sandwich ELISA method (Shenzhen Jingmei Biological Engineering Co., Ltd., Shenzhen, China). A rat anti-human VEGF monoclonal antibody (Shenzhen Jingmei Biological Engineering Co., Ltd.) was coated onto an ELISA plate, and standard and biotinylated detection antibody, double antibody, and specimen and standard VEGF samples were added, which formed an ‘antibody-VEGF-antibody’ compound. The ingredients that did not form part of the compound were washed off. Horseradish peroxidase-labeled avidin and biotin were added to achieve avidin-specific binding. Again, the ingredients that did not combine were washed off and the color reagents, H_2_O_2_ and 3,3′,5,5′-tetramethylbenzidine (Shenzhen Jingmei Biological Engineering Co., Ltd.) were added. If VEGF was present, the reagent turned blue. A terminating agent was added and the reaction color changed to yellow. The optical density (OD) was measured at 450 nm; the level of VEGF is proportionate to OD450, whereby a high density of VEGF results in a low OD. This enabled the calculation of the VEGF concentration of the samples by constructing a standard curve.

### Specimen collection

Ten normal, healthy subjects (five males and five females; mean age, 46.4 years; range, 20–63 years) were selected at random and served as the control group, with 10 individuals with hemangiomas (six males and four females; mean age, 48.8 years) forming the treatment group (pathological classification: Cavernous hemangioma of; the lower lip [n=3], the tongue [n=1], the upper lip [n=3] and of the cheek [n=3]). However, two of the 10 hemangioma patients did not complete the follow-up treatment. The treatment process was as follows: Pure alcohol was injected into the tumors of the hemangioma patients using 5-ml disposable syringes following routine sterilization. When the color of the tumor turned pale the injection was stopped. Each of the 10 hemangioma patients was injected with pure alcoho1 once. Blood samples were obtained prior to the injections, as well as one week and one month after the injections. The blood samples were collected from the patients when they were in a fasted state. Peripheral venous blood (4 ml) was extracted (without anticoagulant) into a centrifuge tube, which was frozen at −112°F in a freezer. One week following the injection of absolute alcohol, the samples were stained with hematoxylin and eosin and observed using a microscope (BX53; Olympus Corporation, Tokyo, Japan). Written informed consent was obtained from all patients and this study was approved by the ethics committee of the Second Affiliated Hospital of Harbin Medical University.

### Experimental procedure

ELISA was used in the present study and the human VEGF kits were purchased from Shenzhen China Crystal Co. Ltd (Shenzhen, China). The double antibody sandwich ELISA method was used, which included the following steps. The cleaning mixture was diluted with deionized water at a ratio of 1:20. The reference material comprised of the following: 1.0 ml Standard/sample diluent was placed into 20 ng of the reference material (at a concentration of 20,000 pg/ml) using a 1,000-μl pipette. The mixture was allowed to dissolve completely for 15 min at room temperature, then the reference material was confected at the following concentrations: 1,000, 500, 250, 125, 62.5, 31.25, and 0 pg/ml. To analyze the blood samples, they were removed from the −112°F freezer and they dissolved completely at room temperature. The blood samples were centrifuged (LD5-10B; Beijing Jingli Centrifuge Co., Ltd., Beijing, China) at a speed of 80 × g for 10 min. The 96-well reaction plates (Shenzhen Jingmei Biological Engineering Co., Ltd.) were coated with the blood samples, and 100 μl standard or sample serum was then added to the corresponding well using a 100-μl micropipette. The plate was incubated at 98.6°F for 90 min and washed four times with prepared washing solution. A total of 200 μl biotinylated antibody working solution (20,000 pg/ml) was added to each well and then the the reaction wells were covered with seal adhesive tape, incubated at 98.6°F for 60 min, and washed four times with the prepared washing solution. Enzyme conjugate (100 μl) was added to the working solution in each well and the reactions were incubated at 98.6°F for 30 min, followed by four sequential washes with the prepared washing solution. The color reagent (100 μl) was added to the working solution in each well, and the reactions were incubated at 98.6°F for 2 min and maintained in the dark. Stopping solution (100 μl) was added to each well and the reaction solution was mixed. The absorbance was then measured using an ELISA analyzer (9606; Perlong Medical Equipment Co., Ltd., Beijing, China) at a wavelength of 450 nm (completed within 5 min) and the results were automatically printed and revealed the OD values (Table I). The results were subsequently converted to units of pg/ml (Table II), and a bilateral t-test was performed as part of the statistical analysis (Table III).

The treatment evaluation criteria included the following: i) The outcome was considered excellent if the tumor completely disappeared, there was normal skin and mucosa, no dysfunction and the tongue movement test was negative; ii) the outcome was considered valid if the tumor regression was >25% (but did not completely disappear), deformity improved, and the posture mobility test was either negative or positive; and iii) the outcome was considered invalid if the tumor regression was <25% or no signs of significant improvement were observed prior to and following treatment.

### Statistical analysis

Data were expressed as the mean ± standard deviation. One-way analysis of variance was performed, and the post hoc Student–Newman–Keuls test was used for multiple comparisons. Statistics were calculated using SPSS version 15.0 for Windows (SPSS Inc., Chicago, IL, USA) and P<0.05 was considered to indicate a statistically significant difference.

## Results

### Characteristic features of a tongue hemangioma

**Figure 1:**
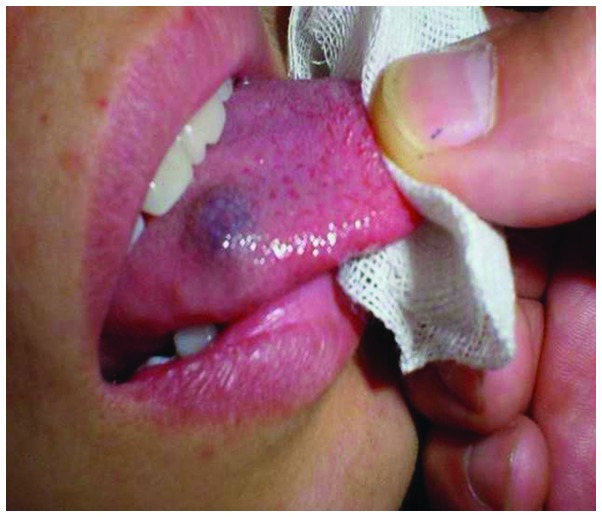
Tongue hemangioma prior to treatment.

**Figure 2:**
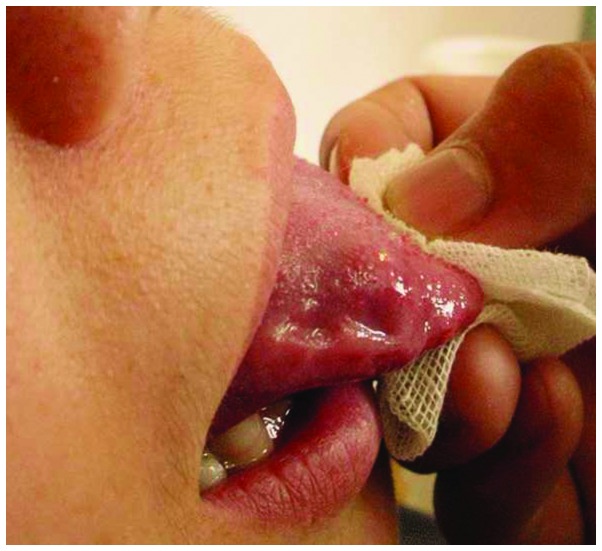
Tongue hemangioma one week following treatment.

**Figure 3:**
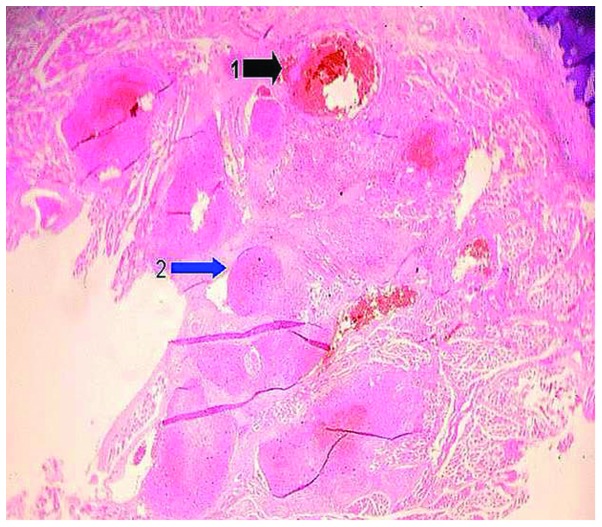
Local pathological changes observed by optical microscopy one week after the injection with pure alcohol. Arrow 1 shows a normal vessel in the hemangioma and arrow 2 shows fibrosis of the local tissue. (stain, hematoxylin and eosin; magnification, ×10).

A tongue hemangioma is demonstrated in Fig. 1. The following characteristics of a tongue hemangioma were observed: Raised mass, purple and soft on palpation. Fig. 2 shows the local pathological changes that occurred one week after an injection of pure alcohol. The local structure was no longer protruding, the mucosal color had returned to normal, the tongue was freely moveable without dysfunction, and the patient’s voice was unchanged. Fig. 3 demonstrates the local pathological changes that were obseved by optical microscopy one week after the injection of pure alcohol. The injection of pure alcohol led to the formation of a thrombus-like structure within the lumen of the hemangioma. Certain regions of the lumen of the hemangioma were blocked completely, whereas other regions were only partially blocked. Such structural characteristics of the lumen ensure a local blood supply and avoid the local formation of necrotic tissue. These factors elucidate the reason that pure alcohol is successful in the treatment of hemangiomas.

### Comparison of serum VEGF levels

The serum VEGF levels of the 10 patients with hemangioma were significantly higher when compared with those of the healthy group (P<0.01). The serum VEGF levels of the 10 hemangioma patients one week after the pure alcohol injections were not significantly different when compared with the values obtained prior to treatment (P<0.01), however, were significantly different from the healthy group. This result was due to VEGF secretion by vascular endothelial cells and also by hematopoietic stem cells, such as platelets, megakaryocytes, monocytes and lymphocytes (7). The increase in the VEGF concentration of the peripheral blood was due to the stimulation of local oxidants, which resulted in the synthesis and secretion of compensatory VEGF (8). The serum VEGF levels of eight of the hemangioma patients one week after the pure alcohol injections were not identified to be significantly different than those of the healthy group. The serum VEGF levels in the peripheral blood were close to normal levels. This result was due to the disappearance of the body’s inflammatory response one month after the pure alcohol injections, which had metabolized the VEGF (9).

## Discussion

Hemangiomas are a type of congenital benign tumor or vascular malformation with the highest incidence rate observed in infants and young children (10). Hemangiomas, particularly facial lesions, may result in significant psychological distress for the parents as well as the patients (11). Hemangiomas occasionally disappear with age; however, even in cases of complete regression, 38% of patients continue to present with residual alterations, such as telangiectasias, yellowish discoloration, scarring or dermal atrophy (12). A subset of hemangiomas require active treatment, with the aim of therapy being the reduction or eradication of the hemangioma while minimizing infection, pain and scarring. Numerous treatment modalities have been reported in the literature, ranging from pharmacological therapies to electroacupuncture (13). Treatment options have expanded to include systemic and intralesional corticosteroids, laser ablation, open submucosal resection, and tracheostomy, with a concomitant reduction in the mortality rate observed of 4% (14).

There are numerous methods for the treatment of hemangiomas, such as surgery, laser treatment, cryotherapy, interferon therapy, hormone therapy, partial radiation therapy, and local injections, as well as others. Pure alcohol injection therapy is frequently used in clinical applications. Currently, there is no objective clinical indicator to assess the therapeutic effects and prognoses following the application of these various treatment strategies. Thus, the aim of the present study was to establish an objective indicator to quantify the therapeutic effect. The exact pathogenesis of hemangiomas remains unclear. The majority of studies hypothesize that VEGF is a direct stimulus for hemangiomas and that the continued elevated expression of it is a key factor in the development of hemangiomas (15,16). VEGF was characterized by Senger *et al* (17) in 1986 and was initially termed the vascular permeability factor (18). In humans, the VEGF-A gene contains a coding region of ~14 kb is on chromosome 6p12. The gene has eight exons that are interspersed with seven introns. Five distinct VEGF protein isoforms have been identified; VEGF121, VEGF145, VEGF165, VEGF189 and VEGF206. Although these VEGF proteins are structurally similar, they are characterized by variations in function and differences in binding specificity. There are important environmental effects of VEGF expression, for example, hypoxia is the most potent agonist of VEGF induction *in vitro* and *in vivo* (19). The expression of VEGF mRNA is induced rapidly and reversibly by hypoxia in numerous cell types, including normal, transformed and tumorigenic cells (20,21). Furthermore, VEGF is a dipolymer composed of two identical subunits (connected via disulfide bonds), with a molecular weight of 17,000–22,000 Da. Depending on alternative splicing during RNA transcription, VEGF can be divided into five subtypes: VEGF121, VEGF145, VEGF165, VEGF189 and VEGF208 (9). There are six VEGF variants, each with similar proteins that are involved in the regulation and differentiation of the vascular system, particularly in the blood and lymph vessels (22). VEGF exerts its effect via VEGF receptors that initially undergo autophosphorylation and subsequently activate phosphatidylcholine-specific phospholipase C (PLC-r). PLC-r hydrolyzes phosphatidylinositol diphosphate, producing diacylglycerol (DAG) and inositol triphosphate. DAG activates protein kinase C, which is present in the cytoplasm, and binds it to the membrane, inducing endothelial cell growth and increasing vascular permeability.

In the present study, the VEGF levels in the hemangioma patient group were significantly higher than those in the healthy group (P<0.01). This finding indicates that the serum concentrations of VEGF increased in the hemangioma patients. The serum VEGF levels of the 10 hemangioma patients one week after the pure alcohol injections were not identified to be significantly different when compared with the levels observed prior to treatment, however, were significantly different from the healthy group. This result occurred as VEGF is expressed by vascular endothelial cells, and by hematopoietic stem cells, such as platelets, megakaryocyte, monocytes and lymphocytes (23). However, VEGF is also expressed by endothelial cells, macrophages, and activated smooth muscle cells in atherosclerotic lesions and is expressed as a result of in-stent restenosis neovascularization (8,24,25). The increase in VEGF concentration in the peripheral blood one week after the injections was due to the stimulation of local oxidants, resulting in the synthesis and secretion of compensatory VEGF. The serum VEGF levels in the peripheral blood had almost returned to baseline (similar to the levels of the control subjects) one month after the pure alcohol injections. This result occurred due to the disappearance of the body’s inflammatory response one month after the pure alcohol injections, resulting in metabolization of excess levels of VEGF.

In conclusion, the clinical effects of local pure alcohol injections into hemangiomas in this study were consistent with previous studies. The ELISA measurements of the serum VEGF concentration in the peripheral blood accurately reflected the concentrations of VEGF in the blood. Therefore, the serum VEGF concentration in the peripheral blood may be used as a clinical indicator of the efficacy of clinical treatment and to determine the prognosis. The present study provides the basis for future scientific research and clinical investigations.

## Figures and Tables

**Table I:** Optical density of patient blood samples measured at a wavelength of 450 nm.

	Optical density									
	
Group	Patient 1	Patient 2	Patient 3	Patient 4	Patient 5	Patient 6	Patient 7	Patient 8	Patient 9	Patient 10
Healthy subjects	0.048	0.057	0.043	0.046	0.048	0.034	0.031	0.052	0.045	0.044
Hemangioma patients	0.337	0.424	0.445	0.365	0.441	0.420	0.351	0.414	0.410	0.400
Post pure alcohol injection										
1 week	0.415	0.455	0.445	0.363	0.350	0.420	0.425	0.401	0.415	0.450
1 month	0.058	0.051	0.050	0.042	0.045	0.059	0.060	0.054		
Control	0.0									

**Table II:** Levels of VEGF (optical density converted to pg/ml).

	VEGF level, pg/ml									
	
Group	Patient 1	Patient 2	Patient 3	Patient 4	Patient 5	Patient 6	Patient 7	Patient 8	Patient 9	Patient 10
Healthy subjects	97.3	115.1	86.5	92.7	95.3	68.3	63.6	103.3	90.2	87.3
Hemangioma patients	650.5	849.3	890.4	730.6	881.3	840.7	701.5	828.2	820.3	800.2
Post pure alcohol injection										
1 week	830.7	909.5	890.4	725.4	700.9	840.8	849.7	801.9	830.8	900.9
1 month	115.4	102.3	100.8	83.5	89.9	118.9	120.7	108.7		
Control	0.0									

VEGF, vascular endothelial growth factor.

**Table III:** VEGF levels in the different groups (presented as the mean ± standard deviation).

Group	Cases, n	VEGF, pg/ml
Healthy subjects	10	97.3±15.1
Hemangioma patients		
Prior to injection	10	799.3±84.1^a^
1 week after injection	10	828.1±68.8^a^
1 month after injection	8	105.6±13.5

a P<0.01 vs. control group.

VEGF, vascular endothelial growth factor.
